# Supramodal neural processing of abstract information conveyed by speech and gesture

**DOI:** 10.3389/fnbeh.2013.00120

**Published:** 2013-09-13

**Authors:** Benjamin Straube, Yifei He, Miriam Steines, Helge Gebhardt, Tilo Kircher, Gebhard Sammer, Arne Nagels

**Affiliations:** ^1^Department of Psychiatry and Psychotherapy, Philipps-University MarburgMarburg, Germany; ^2^Department of General Linguistics, Johannes Gutenberg-University MainzMainz, Germany; ^3^Cognitive Neuroscience at Centre for Psychiatry, Justus Liebig University GiessenGiessen, Germany

**Keywords:** gesture, speech, fMRI, abstract semantics, emblematic gestures, tool-use gestures

## Abstract

Abstractness and modality of interpersonal communication have a considerable impact on comprehension. They are relevant for determining thoughts and constituting internal models of the environment. Whereas concrete object-related information can be represented in mind irrespective of language, abstract concepts require a representation in speech. Consequently, modality-independent processing of abstract information can be expected. Here we investigated the neural correlates of abstractness (abstract vs. concrete) and modality (speech vs. gestures), to identify an abstractness-specific supramodal neural network. During fMRI data acquisition 20 participants were presented with videos of an actor either speaking sentences with an abstract-social [AS] or concrete-object-related content [CS], or performing meaningful abstract-social emblematic [AG] or concrete-object-related tool-use gestures [CG]. Gestures were accompanied by a foreign language to increase the comparability between conditions and to frame the communication context of the gesture videos. Participants performed a content judgment task referring to the person vs. object-relatedness of the utterances. The behavioral data suggest a comparable comprehension of contents communicated by speech or gesture. Furthermore, we found common neural processing for abstract information independent of modality (AS > CS ∩ AG > CG) in a left hemispheric network including the left inferior frontal gyrus (IFG), temporal pole, and medial frontal cortex. Modality specific activations were found in bilateral occipital, parietal, and temporal as well as right inferior frontal brain regions for gesture (G > S) and in left anterior temporal regions and the left angular gyrus for the processing of speech semantics (S > G). These data support the idea that abstract concepts are represented in a supramodal manner. Consequently, gestures referring to abstract concepts are processed in a predominantly left hemispheric language related neural network.

## Introduction

Human communication is distinctly characterized by the ability to convey abstract concepts such as feeling, evaluations, cultural symbols, or theoretical assumptions. This can be differentiated from references to our physical environment consisting of concrete objects and their relationships to each other. In addition to our language capacity, humans also employ gestures as flexible tool to communicate both concrete and abstract information (Kita et al., 2007; Straube et al., 2011a). The investigation of abstractness and modality of communicated information can deliver important insight into the neural representation of concrete and abstract meaning. However, up to now, evidence about communalities or differences in the neural processing of abstract vs. concrete meaning communicated by speech vs. gesture is missing.

Recently, a hierarchical model of language and thought has been suggested (Perlovsky and Ilin, 2010) which proposes that abstract thinking is impossible without speech (Perlovsky and Ilin, 2013). According to this model, abstract information is processed by a neural language system, regardless of whether speech or gesture is chosen as a tool to convey this information. Following this assumption, concrete object-related information is represented in mind independent of speech and hence in a modality-dependent manner in brain regions sensitive to for example visual or motor information. The latter assumption—at least partly—contradicts existing embodiment theories, which suggest a strong overlap of the sensory-motor and language system in particular with respect to the processing of concrete concepts (Gallese and Lakoff, 2005; Arbib, 2008; Fischer and Zwaan, 2008; D'Ausilio et al., 2009; Pulvermüller and Fadiga, 2010). However, the particular role of the communication modality for the neural representation of abstract as opposed to concrete concepts has not been investigated so far.

The impact of abstractness on speech processing (e.g., Rapp et al., 2004, 2007; Eviatar and Just, 2006; Lee and Dapretto, 2006; Kircher et al., 2007; Mashal et al., 2007, 2009; Shibata et al., 2007; Schmidt and Seger, 2009; Desai et al., 2011) and on the neural integration of speech and gesture information has been demonstrated in several functional magnetic resonance imaging (fMRI) studies using different experimental approaches (Cornejo et al., 2009; Kircher et al., 2009b; Straube et al., 2009, 2011a, 2013a; Ibáñez et al., 2011). There is converging evidence suggesting that especially the left inferior frontal gyrus (IFG) plays a decisive role in the processing of abstract semantic figurative meaning in speech (Rapp et al., 2004, 2007; Kircher et al., 2007; Shibata et al., 2007). However, results can further differ due to other factors, such as familiarity, imagibility, figurativeness, or processing difficulty (Mashal et al., 2009; Schmidt and Seger, 2009; Cardillo et al., 2010; Schmidt et al., 2010; Diaz et al., 2011).

In contrast to abstract information processing, it has been suggested that concrete information is processed in different brain regions sensitive to the specific information type: e.g., spatial information in the parietal lobe (Ungerleider and Haxby, 1994; Straube et al., 2011c), form or color information in the temporal lobe (Patterson et al., 2007). A similar finding is illustrated by Binder and Desai (2011): by reviewing 38 imaging studies that examined concrete knowledge processing during language comprehension tasks, the authors found that the processing of action-related speech material activates brain regions that are also involved in action execution (see also Hauk et al., 2004; Hauk and Pulvermüller, 2004); similarly, the processing of other concrete speech information such as sound and color all tend to show activations in areas that process these perceptual modalities (Binder and Desai, 2011). In sum, abstract information processing has been shown to recruit a mainly left-lateralized fronto-temporal neural network whereas concrete information comprehension involves rather diverse activation foci, which are primarily related to the corresponding perceptual origin.

In addition to our speech capacity, gesturing is a flexible communicative tool which humans use to communicate both concrete and abstract information via the visual modality. Previous studies on object- or person-related gesture processing have either presented pantomimes of tool or object use, hands grasping for tools or objects (e.g., Decety et al., 1997; Faillenot et al., 1997; Decety and Grèzes, 1999; Grèzes and Decety, 2001; Buxbaum et al., 2005; Filimon et al., 2007; Pierno et al., 2009; Biagi et al., 2010; Davare et al., 2010; Emmorey et al., 2010; Jastorff et al., 2010); or symbolic gestures like “thumbs up” (Nakamura et al., 2004; Molnar-Szakacs et al., 2007; Husain et al., 2009; Xu et al., 2009; Andric et al., 2013). However, few studies directly compared abstract-social (person-related) with concrete-object-related gestures. A previous study demonstrated that the left IFG is involved in the processing of expressive (emotional) in contrast to body referred and isolated (object-related) hand gestures (Lotze et al., 2006). This finding suggests that the left IFG is sensitive for the processing of abstract information irrespective of communication modality (speech or gestures).

In sum, the left IFG represents a sensitive region for abstract information processing in speech or gesture, whereas the brain areas activated by concrete information depend on communication modality and semantic content. However, whether the same neural structures are relevant for the processing of gestures and sentences with an abstract content or gestures and sentences with a concrete content remains unknown.

Common neural networks for the processing of speech and gesture information have been suggested (Willems and Hagoort, 2007), and empirically tested in several recent studies (Xu et al., 2009; Andric and Small, 2012; Straube et al., 2012; Andric et al., 2013). Andric et al. (2013) performed an fMRI study on gesture processing presenting two different kinds of hand actions (emblematic gestures and grasping movements) and speech to their participants. Thus, either emblematic gestures—hand and arm movements conveying social or symbolic meaning (e.g., “thumbs up” for having done a good job)—or grasping movements (e.g., grasping a stapler) not carrying any semantic meaning *per se* were presented. The authors identified two different types of brain responses for the processing of emblematic gestures: the first type was related to the processing of linguistic meaning, the other type corresponded to the processing of hand actions or movements, regardless of the symbolic meaning conveyed. The latter type involved brain responses in parietal and premotor areas in connection with hand movements, whereas meaning bearing information, e.g., emblem and speech, resulted in activations in left lateral temporal and inferior frontal areas. Altogether, different modalities were involved distinguishing the level of mere perceptual recognition and interpretation of socially and culturally relevant emblematic gestures. More importantly, although lacking baseline conditions containing more concrete semantics (either in gesture or speech), the results from this study tentatively imply a common neural network for processing abstract meaning, irrespective of its input modality.

In a similar vein, Xu et al. (2009) investigated the processing of emblems and pantomimes and their corresponding speech utterances via fMRI. Their finding converges with Andric and colleagues imaging results in the sense that both input modalities activated a common, left-lateralized network encompassing inferior frontal and posterior temporal regions. However, although utilizing emblems (abstract) and pantomimes (concrete) as stimuli, the authors did not elaborate on how different levels of semantics (abstract/concrete) are processed via gesture or speech. Moreover, in a recent study from our laboratory, Straube et al. (2012) looked at less conventionalized gesture—iconic gesture, but still found a fronto-temporal network which was responsible for both the processing of gesture and speech semantics. Altogether, the three aforementioned studies unanimously suggest a common fronto-temporal neural network to be responsible for the processing of not only speech but also gesture semantics.

Although tentative proposals regarding a supramodal neural network for speech and gesture semantics have been made (Xu et al., 2009; Straube et al., 2012), it remains unclear how different levels of semantics—either concrete or abstract—are processed differently with respect to the input modalities. To date, no study results on a direct comparison between abstract and concrete semantic information processing with visual (gesture) or auditory (speech) input are available.

As hypothesized above, concrete object-related information might be represented in mind with and/or without speech, whereas abstract information could require/rely on a representation in speech. Consequently, common processing mechanisms for the processing of speech and gesture semantics can be specifically expected when abstract (in contrast to concrete) information is communicated. Therefore, the current study focused on the neural correlates of abstractness and modality in a communication context. With a factorial manipulation of content (abstract vs. concrete) and communication modality (speech vs. gestures) we wanted to shed light on supramodal neural network properties relevant for the processing of abstract in contrast to concrete information. We tested the following alternative hypotheses: first, if only abstract concepts—activated through speech or gesture in natural communication situations—are processed in a supramodal manner, then we predict consistent neural signatures only for abstract in contrast to concrete contents across different types of communication modality. However, if concrete concepts—activated through speech or gestures—are also represented in a supramodal network, we predict overlapping neural responses for concrete in contrast to abstract contents across modality.

To manipulate abstractness and communication modality we used video clips of an actor either speaking sentences with an abstract-social [AS] or concrete-object-related content [CS], or performing meaningful abstract-social (emblematic) [AG] or concrete-object-related (tool-use) gestures [CG]. Gestures were accompanied by a foreign language (Russian) to increase the comparability between conditions and naturalness of the gesture videos where spoken language frames the communication context. We used emblematic and tool-related gestures to guarantee high comprehensibility of the gestures. During the experiments participants performed a content judgment task referring to the person vs. object-relatedness of the speech and gesture communications to ensure their attention to the semantic information and the adequate comprehension of the corresponding meaning. We hypothesized modality independent activations exclusively for the processing of abstract information (AS > CS ∩ AG > CG) in language-related regions encompassing the left inferior frontal gyrus, the left middle, and superior temporal gyrus (MTG/STG) as well as regions related to social/emotional processing such as the temporal pole, the medial frontal, and anterior cingulate cortex (ACC). In addition, modality specific activations were expected in bilateral occipital, parietal, and temporal brain regions for gesture (G > S) and in left temporal, temporo-parietal, and inferior frontal regions for the processing of speech semantics (S > G).

## Methods

### Participants

Twenty healthy subjects (7 females) participated in the study. The mean age of the subjects was 25.4 years (*SD*: 3.42, range: 22.0–35.0). All participants were right handed (Oldfield, 1971), native German speakers and had no knowledge of Russian. All subjects had normal or corrected-to-normal vision, none reported any hearing deficits. Exclusion criteria were a history of relevant medical or psychiatric illness of the participants. All subjects gave written informed consent prior to participation in the study. The study was approved by the local ethics committee.

### Stimulus material

**Figure 1:**
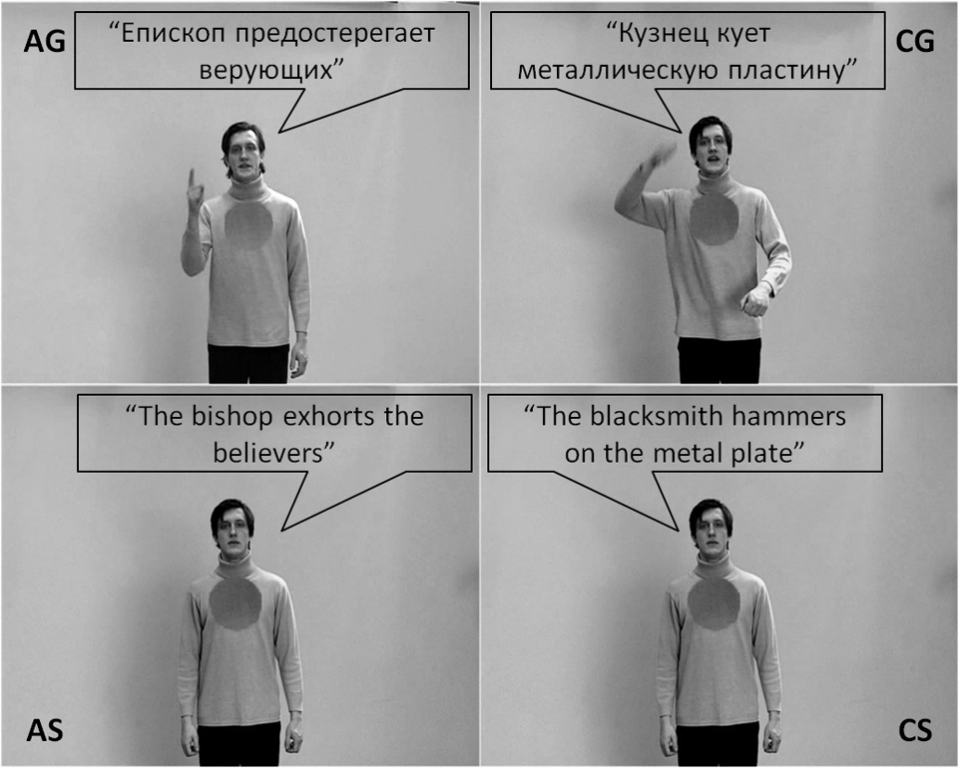
**For each of the four conditions (AG, abstract-gesture; CG, concrete-gesture; AS, abstract-speech; CS, concrete-speech) an example of the stimulus material is depicted**. Note: For illustrative purposes the spoken German sentences were translated into English and all spoken sentences were written into speech bubbles.

Video clips were selected from a large pool of different videos. Some of them have been used in previous fMRI studies, focusing on different aspects of speech and gesture processing (Green et al., 2009; Kircher et al., 2009b; Straube et al., 2009, 2010, 2011a,b, 2012, 2013a,b; Leube et al., 2012; Mainieri et al., 2013). Here, we used emblematic and tool-related gestures and corresponding sentences to guarantee high comprehensibility of the gestures and a strong difference in abstractness between conditions. For the current analysis, 208 (26 videos per condition × 4 conditions × 2 sets) short video clips depicting an actor were used. The actor performed the following conditions: (1) German sentences with an abstract-social content [AS], (2) Russian sentences with abstract-social (emblematic) gestures [AG], (3) German sentences with a concrete-object-related content [CS], and (4) Russian sentences with concrete-object-related (tool-use) gestures [CG] (Figure 1). Thus, we presented videos with semantic information only in speech or only in gesture, both of them in either a highly abstract-social or a concrete-object-related version. Additionally, two bimodal meaningful speech-gesture conditions and one meaningless speech-gesture condition have been presented, which are not of interest for the current analysis.

We decided to present gestures accompanied by a foreign language to increase the comparability between conditions and the naturalness of the gesture videos where spoken language frames the communication context. All sentences had a similar grammatical structure (subject—predicate—object) and were translated into Russian for the gesture conditions. Words that sounded similar in each language were avoided. Examples for the German sentences are: “The blacksmith **hammers** on the metal plate” (“Der Schmied hämmert auf die Metallplatte”; CS condition) or “The bishop **exhorts** the believers” (“Der Bischof ermahnt die Gläubigen”; AS condition; see Figure 1). Thus, the sentences had a similar length of five to eight words and a similar grammatical form, but differed considerably in content. The corresponding gestures (keyword indicated in bold) matched the corresponding speech content, but were presented here only in a foreign language context.

The same male bilingual actor (German and Russian) performed all the utterances and gestures in a natural spontaneous way. Intonation, prosody and movement characteristics in the corresponding variations of one item were closely matched. At the beginning and at the end of each clip the actor stood with arms hanging comfortably. Each clip had a duration of 5 s including 500 ms before and after the experimental manipulation, where the actor neither spoke nor moved. In the present study the semantic aspects of the stimulus material refer to differences in abstractness of the communicated information (abstract vs. concrete content).

For stimulus validation, 20 participants not taking part in the fMRI study rated each video on a scale from 1 to 7 concerning understandability, imageability and naturalness (1 = very low to 7 = very high). In order to assess *understandability* participants were asked: How understandable is the video clip? (original: “Wie VERSTÄNDLICH ist dieser Videoclip?”). The rating scale ranged from 1 = very difficult to understand (sehr schlecht verständlich) to 7 = very easy/good to understand (sehr gut verständlich). For *naturalness* ratings the participants were asked: How natural is the scene? (original: “Wie NATÜRLICH ist diese Szene?”). The rating scale ranged from 1 = very unnatural (sehr unnatürlich) to 7 = very natural (sehr natürlich). Finally, for judgments of *imageability* the participants were asked: How pictorial/imageable is the scene? (original: “Wie BILDHAFT ist dieser Videoclip?”). The rating scale ranged from 1 = very abstract (sehr abstrakt) to 7 = very pictoral/imageable (sehr bildhaft). These scales have been used in previous investigations, too (Green et al., 2009; Kircher et al., 2009b; Straube et al., 2009, 2010, 2011a,b). A set of 338 video clips (52 German sentences with concrete-object-related content, 52 German sentences with abstract-social content and their counterparts in Russian-gesture and German-gesture condition and 26 Russian control condition) were chosen as stimuli for the fMRI experiment on the basis of high naturalness and high understandability for the German and gesture conditions. The stimuli were divided into two sets in order to present each participant with 182 clips during the scanning procedure (26 items per condition), counterbalanced across subjects. A single participant only saw complementary derivatives of one item, i.e., the same sentence or gesture information was only presented once per participant. This was done to avoid speech or gesture repetition or carryover effects. Again, all parameters listed above were used for an equal assignment of the video clips to the two experimental sets, to avoid set-related between-subject differences. As an overview, Table 1 lists the mean durations of speech and gestures as well as the mean ratings of comprehension, imageability, and naturalness of the items used for the current analyses.

**Table 1:** **Number of videos and their mean durations of stimulus parameters speech and gesture as well as their mean stimulus ratings of understandability, imageability, and naturalness according to the four conditions abstract-gesture (AG), concrete-gesture (CG), abstract-speech (AS), and concrete-speech (CS) for set 1, set 2 and in total**.

**Set**	**Condition**	***N***	**Stimulus parameter**				**Rating evaluations**					
			**Speech duration**		**Gesture duration**		**Understandability**		**Imagebility**		**Naturalness**	
			**Mean**	***SD***	**Mean**	***SD***	**Mean**	***SD***	**Mean**	***SD***	**Mean**	***SD***
1	AG	26	2.163	0.391	2.313	0.440	3.625	0.578	4.498	0.587	4.565	0.379
	CG	26	2.303	0.434	3.033	0.364	3.537	0.808	4.785	0.695	4.340	0.540
	AS	26	2.400	0.308			6.527	0.179	3.481	0.321	4.077	0.258
	CS	26	2.332	0.290			6.650	0.209	2.967	0.308	3.181	0.293
	Total	104	2.299	0.366	2.673	0.540	5.085	1.595	3.933	0.894	4.041	0.649
2	AG	26	2.144	0.296	2.219	0.336	3.392	0.766	4.381	0.698	4.479	0.501
	CG	26	2.160	0.391	2.989	0.415	3.327	0.660	4.598	0.621	4.181	0.444
	AS	26	2.332	0.281			6.490	0.154	3.454	0.372	3.935	0.237
	CS	26	2.274	0.229			6.652	0.155	3.083	0.207	3.181	0.279
	Total	104	2.228	0.311	2.604	0.539	4.965	1.693	3.879	0.810	3.944	0.612
Total	AG	52	2.153	0.343	2.266	0.390	3.509	0.682	4.439	0.641	4.522	0.442
	CG	52	2.231	0.415	3.011	0.387	3.432	0.738	4.691	0.659	4.261	0.496
	AS	52	2.366	0.294			6.509	0.166	3.467	0.344	4.006	0.256
	CS	52	2.303	0.260			6.651	0.182	3.025	0.266	3.181	0.283
	Total	208	2.263	0.340	2.639	0.538	5.025	1.642	3.906	0.851	3.992	0.631

SD, standard deviation.

The ratings on understandability for the videos of the four conditions used in this study clearly show a main effect of modality, with the speech varieties scoring higher than the gesture varieties [*F*_(1, 113.51)_ = 1878.79, *P* < 0.001, two-factorial between-subjects ANOVA with adjusted degrees of freedom according to Brown–Forsythe]. This effect stems from the fact that different languages were used for speech only and gesture with speech conditions. Video clips with German speech scored higher than 6 while Russian speech with gestures videos scored between 3 and 4 (6.58 vs. 3.47, respectively). This difference is in line with the assumption that when presented without the respective sentence context isolated gestures are less meaningful, but even then they still are more or less understandable, which was important for the current study.

Imageability ratings indicated that there were also differences between the conditions concerning their property to evoke mental images. A significant main effect for modality showed that videos consisting of Russian sentences with gesture were evaluated as being better imaginable than videos consisting only of German sentences [4.57 vs. 3.25, respectively; *F*_(1, 144.92)_ = 349.89, *P* < 0.001, two-factorial between-subjects ANOVA with adjusted degrees of freedom according to Brown–Forsythe]. A significant interaction effect indicated that this difference was even more pronounced for the concrete conditions [*F*_(1, 144.92)_ = 24.22, *P* < 0.001, two-factorial between-subjects ANOVA with adjusted degrees of freedom according to Brown–Forsythe].

Naturalness ratings showed a main effect for modality as well. Videos including Russian sentences with gestures were evaluated as more natural than videos including German speech [4.39 vs. 3.59, respectively; *F*_(1, 160.63)_ = 225.65, *P* < 0.001, two-factorial between-subjects ANOVA with adjusted degrees of freedom according to Brown–Forsythe]. There was also a difference in naturalness ratings concerning the abstractness of the included content. Videos depicting concrete content were evaluated as being less natural than videos depicting abstract content [4.26 vs. 3.72, respectively; *F*_(1, 160.63)_ = 104.48, *P* < 0.001, two-factorial between-subjects ANOVA with adjusted degrees of freedom according to Brown–Forsythe]. Additionally, an interaction effect indicated that videos consisting of German speech with concrete content were evaluated as least natural [*F*_(1, 160.63)_ = 28.18, *P* < 0.001, two-factorial between-subjects ANOVA with adjusted degrees of freedom according to Brown–Forsythe].

The sentences had an average speech duration of 2263 ms (*SD* = 340 ms), with German sentences being somewhat longer than Russian sentences [2335 vs. 2192 ms, respectively; *F*_(1, 180.94)_ = 9.51, *P* < 0.05, two-factorial between-subjects ANOVA with adjusted degrees of freedom according to Brown–Forsythe]. The gestures analyzed here had an average gesture duration of 2639 ms (*SD* = 538 ms), with gestures for concrete content being longer than gestures for abstract content [3011 vs. 2266 ms, respectively; *T*_(102)_ = 9.78, *P* < 0.001].

Events for the fMRI statistical analysis were defined in accordance with the bimodal German conditions [compare for example Green et al. (2009); Straube et al. (2012)] as the moment with the highest semantic correspondence between speech and gesture stroke (peak movement): Each sentence contained only one element that could be illustrated, which was intuitively done by the actor. The events occurred on average 2036 ms (*SD* = 478 ms) after the video start and were used for the modulation of events in the event-related fMRI analysis. The use of these predefined integration time points (see Green et al., 2009) for the fMRI data analysis had the advantage that the timing for all conditions of one stimulus was identical since conditions were counterbalanced across subjects. Additionally, speech and gesture duration were used as parameters of no interest on single trial level to control for condition specific differences in these parameters.

### Experimental procedure

During fMRI data acquisition participants were presented with videos of an actor either speaking sentences (S) or performing meaningful gestures (G) with an abstract-social (A) or concrete-object-related (C) content. Gestures were accompanied by an unknown foreign language (Russian). Participants performed a content judgment task referring to the person vs. object-relatedness of the utterances.

### fMRI data acquisition

All MRI data were acquired on a 3T scanner (Siemens MRT Trio series). Functional images were acquired using a T2-weighted echo planar image sequence (*TR* = 2 s, *TE* = 30 ms, flip angle 90°, slice thickness 4 mm with a 0.36 mm interslice gap, 64 × 64 matrix, FoV 230 mm, in-plane resolution 3.59 × 3.59 mm, 30 axial slices orientated parallel to the AC-PC line covering the whole brain). Two runs of 425 volumes were acquired during the experiment. The onset of each trial was synchronized to a scanner pulse.

### Experimental design and procedure

An experimental session comprised 182 trials (26 for each condition) and consisted of two 14-min blocks. Each block contained 91 trials with a matched number of items from each condition (13). The stimuli were presented in an event-related design in pseudo-randomized order and counterbalanced across subjects. As described above (stimulus material) across subjects each item was presented in corresponding conditions, but a single participant only saw complementary derivatives of one item, i.e., the same sentence or gesture information was only seen once per participant. Each clip was followed by a gray background with a variable duration of 2154–5846 ms (jitter average: 4000 ms).

Before scanning, each participant received at least six practice trials outside the scanner to ensure comprehensive understanding of the experimental task. Prior to the start of the experiment, the volume of the videos was individually adjusted so that the clips were clearly audible. During scanning, participants were instructed to watch the videos and to indicate via left hand key presses whether the content of the sentence or the gesture referred to objects index finger or interpersonal social information (e.g., feelings, requests, etc.) middle finger. This task enabled us to focus participants' attention to the semantic content of speech and gesture and to investigate comprehension in a rather implicit manner. Performance rates and reaction times were recorded.

### MRI data analysis

MR images were analyzed using Statistical Parametric Mapping (SPM8) standard routines and templates (www.fil.ion.ucl.ac.uk). After discarding the first five volumes to minimize T1-saturation effects, all images were spatially and temporally realigned, normalized (resulting voxel size 2 × 2 × 2 mm^3^), smoothed (8 mm isotropic Gaussian filter) and high-pass filtered (cut-off period 128 s).

Statistical whole-brain analysis was performed in a two-level, mixed-effects procedure. In the first level, single-subject BOLD responses were modeled by a design matrix comprising the onsets of each event within the videos (see stimulus material) of all seven experimental conditions. As additional factor each video phase was modeled as mini-bock with 5 s duration. To control for condition specific differences in speech and gesture duration these stimulus characteristics were used as parameters of no interest on single trial level. The hemodynamic response was modeled by the canonical hemodynamic response function (HRF). Parameter estimate (β-) images for the HRF were calculated for each condition and each subject. Parameter estimates for the four relevant conditions were entered into a within-subject flexible factorial ANOVA.

A Monte Carlo simulation of the brain volume was employed to establish an appropriate voxel contiguity threshold (Slotnick and Schacter, 2004). This correction has the advantage of higher sensitivity to smaller effect sizes, while still correcting for multiple comparisons across the whole brain volume. Assuming an individual voxel type I error of *P* < 0.001, a cluster extent of 50 contiguous resampled voxels was indicated as necessary to correct for multiple voxel comparisons at *P* < 0.05. This cluster threshold (based on the whole brain volume) has been applied to all contrasts. The reported voxel coordinates of activation peaks are located in MNI space. For the anatomical localization, functional data were referenced to probabilistic cytoarchitectonic maps (Eickhoff et al., 2005) and the AAL toolbox (Tzourio-Mazoyer et al., 2002).

### Contrasts of interest

The neural processing of abstract information was isolated by computing the difference contrast of abstract-social vs. concrete-object-related sentences [AS > CS] and gestures [AG > CG], whereas the opposite contrasts were applied to reveal brain regions sensitive for the processing of concrete information communicated by speech [CS > AS] and gesture [CG > AG].

In order to find regions that are commonly activated by both processes, contrasts were entered into a conjunction analysis (abstract: [AS > CS ∩ AG > CG]; concrete: [CS > AS ∩ CG > AG]), testing for independently significant effects compared at the same threshold (conjunction null, see Nichols et al., 2005).

The identical approach has been applied to demonstrate the effect of modality by calculating the following conjunctional analyses, for gesture [AG > AS ∩ CG > CS] and for speech semantics [AS > AG ∩ CS > CG].

Finally, interaction analyses were performed ([AS vs. AG] vs. [CS vs. CG]) to explore modality specific effects with regard to the processing of abstract vs. concrete information. Masking procedure has been used to ensure that all interactions are based on significant differences of the first contrast (e.g., [CG > CS] > [AG > AS] inclusively masked by [CG > CS]).

## Results

### Behavioral results

Subjects were instructed to indicate via button press whether the actor in the video described a socially related action or an object-related action. Correct responses and their reaction times were analyzed each with a Two-Way within-subjects ANOVA with the repeated measurement factors modality (gesture vs. speech) and abstractness (abstract vs. social).

**Figure 2:**
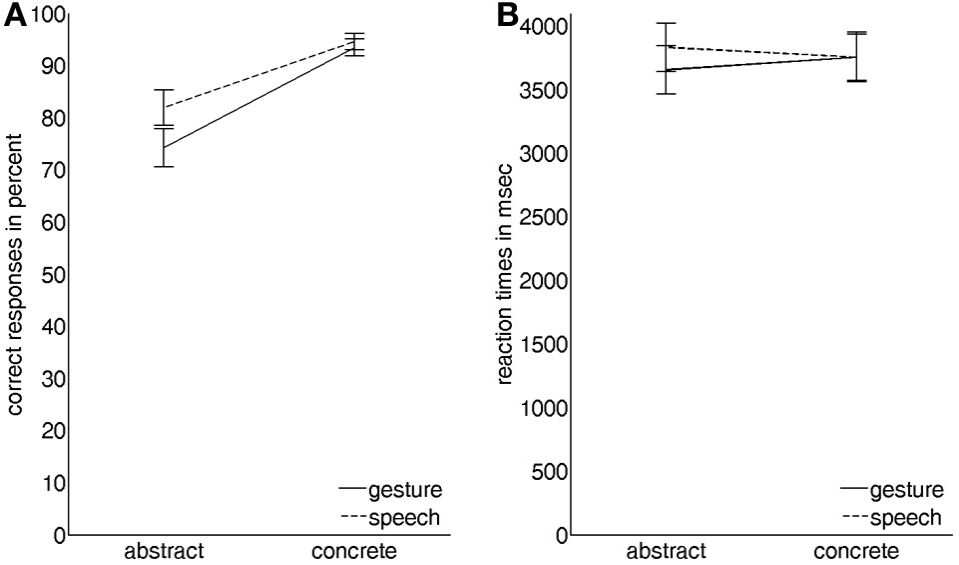
**Graphical illustration of the interaction effects of the two factors modality (gesture vs. speech) and abstractness (abstract vs. concrete) on (A) the number of correct responses in percent and on (B) the corresponding reaction times in ms (vertical lines indicate standard errors of the mean)**.

Correct responses showed a significant main effect for modality with videos depicting gesture with Russian speech receiving slightly lower scores than videos depicting German speech only [21.8 vs. 22.95 out of 26, respectively; *F*_(1, 19)_ = 8.369, *P* < 0.05, partial-eta-squared = 0.31]. A significant main effect for abstractness clearly indicated that videos describing abstract social content were less often identified correctly than videos showing concrete object-related content [20.3 vs. 24.45 out of 26, respectively; *F*_(1, 19)_ = 15.361, *P* < 0.001, partial-eta-squared = 0.45]. The factors modality and abstractness also showed a modest significant interaction effect on correct responses [*F*_(1, 19)_ = 4.572, *P* < 0.05, partial-eta-squared = 0.19] stemming from the fact that for videos depicting abstract content the difference between gesture with Russian speech and German speech was more pronounced than for videos showing concrete object-related content (Figure 2A).

For each participant the median reaction time for each condition was computed from all correct responses of that condition. A significant interaction effect of modality and abstractness [*F*_(1, 19)_ = 5.227, *P* < 0.05, partial-eta-squared = 0.22] indicated that while there was no difference for videos depicting concrete content, participants reacted slightly faster to videos depicting abstract content with gesture and slightly slower to videos of abstract content with German speech (Figure 2B).

### fMRI results

#### Effects of modality

**Figure 3:**
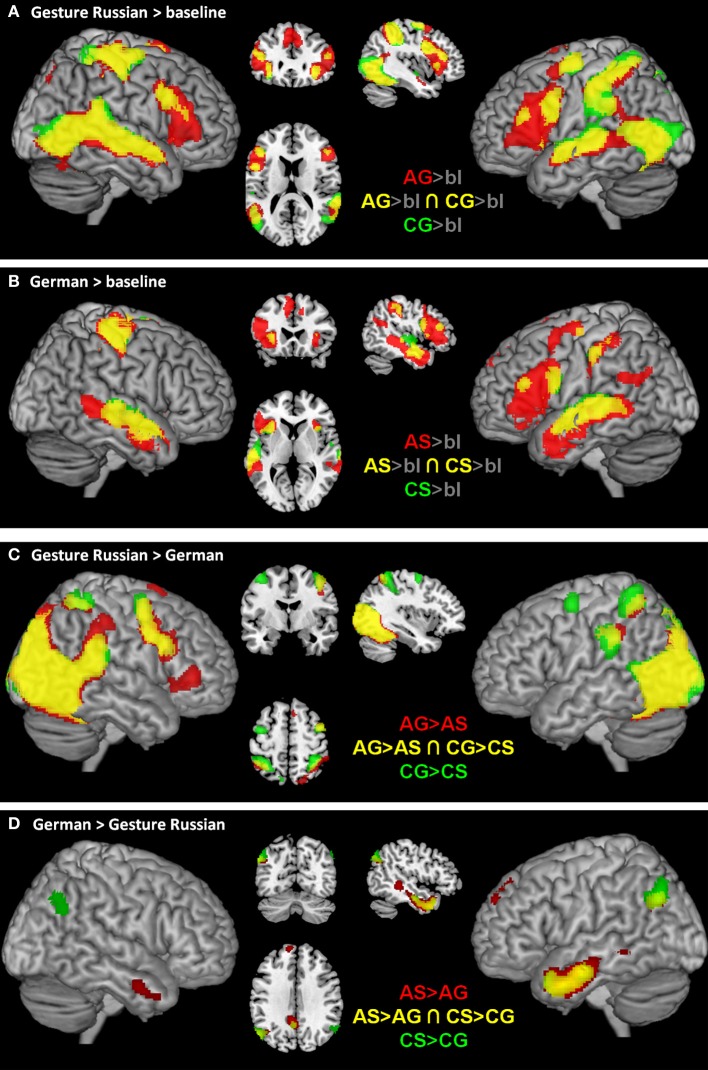
**Illustrates the fMRI results for abstract semantics (red), concrete semantics (green), and common neural structures (yellow) for each condition in contrast to low-level baseline (gray background; A, Gesture; B, German), for gesture conditions in contrast to German conditions (C) and for German in contrast to the gesture conditions (D)**. Results were rendered on brain slices and surface using the MRIcron toolbox (http://www.mccauslandcenter.sc.edu/mricro/mricron/install.html).

For the effect of gesture in contrast to speech semantics independent of the abstractness [AG > AS ∩ CG > CS] we found activation in bilateral occipital, parietal, and right frontal brain regions (see Table 2, and Figure 3C, yellow). By contrast, for the processing of speech semantics independent of abstractness [AS > AG ∩ CS > CG] we found activations in the left anterior temporal lobe and the supramarginal gyrus (see Table 2, and Figure 3D, yellow).

**Table 2:** **Activation peaks and anatomical regions comprising activated clusters for the conjunction contrasts representing effects of modality (speech vs. gesture and vice versa)**.

**Contrast**	**Anatomical regions/hem**.	**No. voxels**	**Peak MNI coordinates**			***t*-value**
			***x***	***y***	***z***	
AS > AG ∩ CS > CG	Middle temporal gyrus L	673	−52	−12	−20	5.61
	Middle temporal pole L					
	Angular gyrus L	166	−54	−68	34	4.81
	Precuneus L	69	−4	−56	34	3.78
AG > AS ∩ CG > CS	Middle occipital gyrus L	6691	−48	−74	4	19.91
	Inferior temporal gyrus L					
	Middle temporal gyrus R	9536	50	−62	0	19.62
	Fusiform gyrus R					
	Superior occipital gyrus R					
	IFG, pars opercularis R	1313	44	10	28	6.72
	Middle frontal gyrus R					
	Precentral gyrus R					
	Supramarginal gyrus L	202	−62	−36	32	4.56
	Superior parietal lobe L	299	−38	−54	60	4.22
	Inferior parietal lobe L					

Table lists the respective contrast, anatomical regions, cluster size, MNI coordinates, and t-values for each significant activation (p < 0.05 corrected for multiple comparisons). MNI, Montreal Neurological Institute; AS, abstract speech; AG, abstract gesture; CS, concrete speech; CG, concrete gesture; IFG, inferior frontal gyrus; L, left; R, right.

The exploration of general activation for each condition in contrast to low-level baseline (gray background) indicates that other regions are commonly activated in all conditions (Figures 3A,B). Most interestingly, the IFG seems to be activated bilaterally in the gesture conditions (Figure 3A) and left lateralized in the speech conditions (Figure 3B).

#### Within modality effects of abstractness

**Figure 4:**
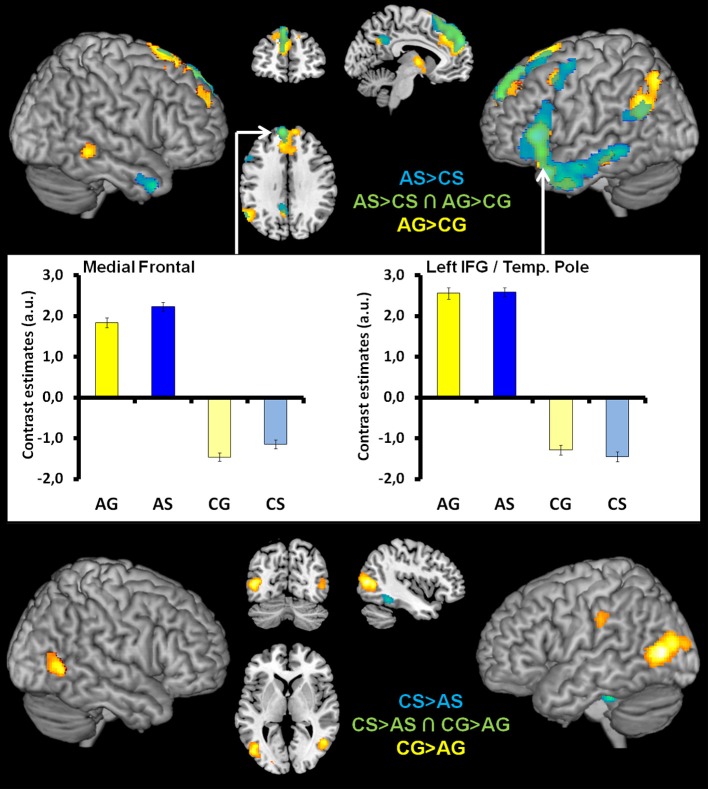
**Top illustrates the within-modality processing of abstractness in language semantics ([AS > CS], blue), gesture semantics ([AG > CG], yellow), and in common neural structures (green, overlapping regions)**. Bar graphs in the **middle** of figure illustrate the contrast estimates (extracted eigenvariates) for the commonly activated (green) medial superior frontal (left) and temporal pole/IFG cluster (right). These are representative for all overlapping activation clusters. The within-modality processing of concrete in contrast to abstract language semantics ([CS > AS], blue) and gesture semantics ([CG > AG], yellow) is illustrated at the **bottom** of figure. Here we found no overlap between activation patterns.

Analyses targeting at within-modality processing of abstractness in language semantics [AS > CS] showed activation in a mainly left-lateralized network encompassing an extended fronto-temporal cluster (IFG, precentral gyrus, middle, inferior, and superior temporal gyrus) as well as medial frontal regions and the right anterior middle temporal gyrus (Table 3 and Figure 4 top, blue). We obtained a comparable activation pattern for the within-modality processing of abstractness in gesture semantics ([AG > CG] see Figure 4 top, yellow). The opposite contrasts revealed activation in clusters encompassing the left cerebellum, fusiform, and inferior temporal gyrus in the language contrast (CS > AS; see Figure 4 bottom, blue) and the bilateral occipital lobe for the gesture contrast (CG > AG; see Figure 4 bottom, yellow).

**Table 3:** **Activation peaks and anatomical regions comprising activated clusters for the contrasts representing effects of abstractness (abstract vs. concrete and vice versa) dependent of modality (speech or gesture)**.

**Contrast**	**Anatomical regions/hem**.	**No. Voxels**	**Peak MNI coordinates**			***t*-value**
			***x***	***y***	***z***	
AS > CS	Middle temporal gyrus L	3150	−52	−34	−6	5.98
	IFG, pars orbitalis L					
	Medial superior frontal gyrus L	1441	−8	56	34	5.72
	Middle temporal pole R	289	48	12	−34	5.16
	Middle temporal gyrus R					
	Angular gyrus L	458	−42	−58	24	4.41
	Precentral gyrus L	248	−38	0	62	4.36
	Precuneus L	195	−8	−50	34	4.22
AG > CG	Superior temporal pole L	910	−36	18	−24	5.43
	IFG, pars triangularis L					
	IFG, pars orbitalis L					
	Medial superior frontal gyrus L	3215	−4	30	54	5.10
	Angular gyrus L	682	−60	−60	30	4.64
	Caudate nucleus R	786	12	2	8	4.54
	Thalamus L					
	Middle temporal gyrus L	209	−48	−16	−18	4.04
AS > CS ∩ AG > CG	Medial superior frontal gyrus L	1015	−8	56	30	4.97
	Superior temporal pole L	779	−36	18	−22	4.93
	IFG, pars triangularis L					
	IFG, pars orbitalis L					
	Middle temporal gyrus L	161	−48	−14	−20	3.99
	Angular gyrus L	253	−54	−56	26	3.95
CS > AS	Cerebellum L	580	−32	−36	−28	5.95
	Inferior temporal gyrus L					
	Fusiform gyrus L					
CG > AG	Middle occipital gyrus L	1046	−44	−76	8	5.86
	Middle temporal gyrus R	285	50	−62	2	4.73
CS > AS ∩ CG > AG						n.s.

Table lists the respective contrast, anatomical regions, cluster size, MNI coordinates, and t-values for each significant activation (p < 0.05 corrected for multiple comparisons). MNI, Montreal Neurological Institute; AS, abstract speech; AG, abstract gesture; CS, concrete speech; CG, concrete gesture; IFG, inferior frontal gyrus; L, left; R, right.

#### Common activations for abstractness contained in gestures and spoken language

Processing of abstract information independent of input modality as disclosed by the conjunction of [AS > CS ∩ AG > CG] was related to a left-sided frontal cluster including the temporal pole, the IFG (pars triangularis and orbitalis), the middle temporal and angular as well as the medial superior frontal gyrus (Table 3 and Figure 4 top middle/right, green). The opposite conjunction analyses [CS > AS ∩ CG > AG] revealed no significant common activation for the processing of concrete in contrast to abstract information.

#### Interaction

No significant activation could be identified in the interaction analyses on the selected significance threshold. However, by applying a different cluster size to voxel level threshold proportion to correct for multiple comparisons (*p* < 0.005 and 86 voxels) as indicated by an additional Monte Carlo simulation, we found an interaction in occipital (MNI *x, y, z*: −20, −90, −8, *t* = 3.63, *p* < 0.001, 140 voxels), parietal (MNI *x, y, z*: −34, −48, 68, *t* = 3.80, *p* < 0.001, 143 voxels; MNI *x, y, z*: −34, −40, 48, *t* = 3.11, *p* < 0.001, 88 voxels) and premotor (MNI *x, y, z*: −34, −4, 62, *t* = 3.55, *p* < 0.001, 129 voxels) regions reflecting an specific increase of activation in these regions for the processing of concrete-object-related gesture meaning ([CG > CS] > [AG > AS] inclusively masked by [CG > CS]).

## Discussion

We hypothesized that the processing of abstract semantic information of spoken language and symbolic emblematic gestures is based on a common neural network. Our study design tailored the comparison to the level of abstract semantics, controlling for processing of general semantic meaning of speech and gesture by using highly meaningful concrete object-related information as control condition. The results demonstrate that the pathways engaged in the processing of semantics contained in both abstract spoken language and abstract-social gestures comprise the temporal pole, the IFG (pars triangularis and orbitalis), the middle temporal, angular and the superior frontal gyri. Thus, in line with our hypothesis we found modality-independent activation in a left hemispheric fronto-temporal network for the processing of abstract information. The strongly left lateralized activation pattern supports the theory that abstract semantics is independent of communication modality represented in language (at least on neural level represented in language-related brain regions).

### Effects of modality

The results of the speech [CS > CG ∩ AS > AG] and gesture contrasts [CG > CS ∩ AG > AS] clearly demonstrate that communication modality affects neural processing in the brain independent of the communication content (abstract/concrete). In line with other studies that contrasted the processing of a native against an unknown foreign language (Perani et al., 1996; Schlosser et al., 1998; Pallier et al., 2003; Straube et al., 2012), we found activation along the left temporal lobe (including STG, MTG, and ITG) for German speech contrasted with Russian speech and gesture. This strongly left-lateralized pattern has been found in all of the above mentioned studies. Apart from these studies with conditions very similar to ours, temporal as well as inferior frontal regions have been frequently implicated in various language tasks (for reviews see Bookheimer, 2002; Vigneau et al., 2006; Price, 2010). The lack of IFG activation in our study is probably dependent on the fact that we compared a native language (CS, AS) with a foreign language which was accompanied by a meaningful gesture (CG, AG). Thus, motoric or semantic processes of the left IFG might be equally involved in the speech and gesture conditions as indicated by baseline contrasts (see Figures 3A,B).

In line with studies on action observation (e.g., Decety et al., 1997; Decety and Grèzes, 1999; Grèzes and Decety, 2001; Filimon et al., 2007) and co-verbal gesture processing (e.g., Green et al., 2009; Kircher et al., 2009b; Straube et al., 2011a), we found for the processing of gesture in contrast to speech information a bilaterally distributed network of activation including occipital, parietal, posterior temporal, and right frontal brain regions.

### Supramodal processing of abstract semantics of speech and gesture

The processing of abstract spoken language semantics (AS > CS) and abstract semantic information conveyed through abstract-social in contrast to concrete-object-related gestures (AG > CG) activated an overlapping network of brain regions. These include a cluster in the left inferior frontal cortex (BA 44, 45) which expanded into the temporal pole, the left inferior, and middle temporal gyrus as well as a cluster in the left medial superior frontal gyrus. Those findings support the model of a supramodal semantic network for the processing of abstract information. By contrast, for concrete vs. abstract information we obtained no overlapping activation.

These results extend studies from both the gesture and the language domain (see above) in showing a common neural representation of specific speech and gesture semantics. Furthermore, the findings go beyond previous reports about common activation for symbolic gestures and speech semantics (Xu et al., 2009), in showing a specific effects for abstract but not concrete speech and gesture information. Interestingly, we previously found similar activation of the left IFG and temporal brain regions for the processing of concrete speech and gesture semantics of iconic gestures (Straube et al., 2012). Whereas iconic gestures are not symbolic and usually occur in a concrete sentence context (e.g., “The ball is round,” using both hands to indicate a round shape), they might implicate rather abstract information without speech, since any concrete meaning can be revealed from these iconic gestures in this context. Thus, the left IFG activation in our previous study could also be explained by an abstract interpretation of isolated iconic gestures (Straube et al., 2012).

The left-lateralization of our findings is congruent with the majority of fMRI studies on language (see Bookheimer, 2002; Price, 2010, for reviews). Left fronto-temporal activations have been frequently observed for semantic processing [e.g., Gaillard et al., 2004; for a review see Vigneau et al. (2006)], the decoding of meaningful actions (e.g., Decety et al., 1997; Grèzes and Decety, 2001) and also with regard to co-verbal gesture processing (Willems et al., 2007, 2009; Holle et al., 2008, 2010; Kircher et al., 2009b; Straube et al., 2011a).

With regard to the inferior frontal activations, functional imaging studies have underlined the importance of this region in the processing of language semantics. The junction of the precentral gyrus and the pars opercularis of the left IFG has been involved in controlled semantic retrieval (Thompson-Schill et al., 1997; Wiggs et al., 1999; Wagner et al., 2001), semantic priming (Sachs et al., 2008a,b, 2011; Kircher et al., 2009a; Sass et al., 2009a,b) and a supramodal network for semantic processing of words and pictures (Kircher et al., 2009a). The middle frontal gyrus (MFG) was found activated by intramodal semantic priming (e.g., Tivarus et al., 2006). However, medial frontal activation in our study might be better explained by differences in social-emotional content between conditions, which have been often found for social functioning, social cognition, theory of mind, or mentalizing (e.g., Uchiyama et al., 2006, 2012; Krach et al., 2009; Straube et al., 2010).

Since semantic memory represents the basis of semantic processing, an amodal semantic memory (Patterson et al., 2007) is a likely explanation for how speech and gesture semantics could activate a common neural network. Our findings suggest supramodal semantic processing in regions including the left temporal pole, which has been described as best candidate for a supramodal semantic “hub” (Patterson et al., 2007). Thus, abstract semantic information contained in speech and gestures might have activated supramodal semantic knowledge in our study more strongly than concrete information communicated by speech and gesture.

Our data also partially coincide with Binder and Desai's (2011) neuroanatomical model of semantic processing: in this model, low level (concrete) sensory, action and emotion semantics are processed in brain areas that are located near corresponding perceptual networks; higher-level semantics (abstract semantics), on the contrary, converges at temporal, and inferior parietal regions (Binder and Desai, 2011). Additionally, as a next step, inferior prefrontal cortices are responsible for the selection of the information stored in temporo-parietal cortices. In the current experiment, abstract information activates both temporal and inferior frontal cortices, and this could be considered as evidence supporting the role of fronto-temporal pathways in the processing of higher-level semantics. More importantly, our results suggest that this processing of abstract information is independent of input modality.

As for the processing of concrete semantics, our results are somewhat surprising because we did not find an overlap between gestural and verbal-auditory input. This result falls beyond the prediction of both strict embodiment theories (Barsalou, 1999; Gallese and Lakoff, 2005; Pulvermüller and Fadiga, 2010) and theories which propose less strict embodiment: all these theories would predict that the concrete semantics in our experiment, being predominantly action-driven, would activate motoric brain regions such as (pre-)motor and parietal cortices, and this activation pattern should be independent of the input modality. However, previous support for these theories is based on studies using single words (e.g., Willems et al., 2010; Moseley et al., 2012) instead of sentences, which might increase the task effort and specifically trigger motoring simulation. Thus, one explanation for the discrepancy between studies could be that we investigated the processing of tool-use information in a sentence context (see Tremblay and Small, 2011). Here, motoric simulation might not be necessary since contextual information facilitates semantic access (e.g., the blacksmith primes the hammer).

Our results are also in line with a recent mathematically-motivated language-cognition model proposed by Perlovsky and Ilin (2013). This model suggests that high-level abstract thinking relies on the language system and low-level and concrete thinking does not necessarily have to. Transferred to a neural perspective, both abstract meaning (irrespective of input modality) and language (processing) would recruit similar neural networks. In our experiment, the left-lateralized network for abstract meaning comprehension fits perfectly to this prediction. Although it still remains unclear how language and higher-level thinking are related at a functional level, our study provides initial neural evidence, which closely connects the two different domains.

## Conclusion

Language is not only a communication device, but also a fundamental part of cognition and learning concepts, especially with respect to abstract concepts (Perlovsky and Ilin, 2013). In the last years the understanding of speech and gesture processing has increased; both communication channels have been disentangled and brought together again. Here we investigated the neural correlates of abstractness (abstract vs. concrete) and modality (speech vs. gestures), to demonstrate the existence of an abstractness specific supramodal neural network.

In fact, we could demonstrate the activation of a supramodal network for abstract speech and abstract gestures semantics. The identified left lateralized fronto-temporal network not only maps sound patterns and their corresponding abstract meanings in the auditory domain, but also combines gestures and their abstract meanings in the gestural-visual domain. This modality-independent network most likely gets input from modality-specific areas in the superior temporal (speech) and occipito-temporal brain regions (gestures), where the main characteristics of the spoken and gestured signals are decoded. The inferior frontal regions are responsible for the process of selection and integration, relying on more general world knowledge distributed throughout the brain (Xu et al., 2009). The challenge for future studies will be the identification of specific aspects of speech and gesture semantics or the respective format relevant for the understanding of natural receptive and productive communicative behavior and its dysfunctions in patients, for example with schizophrenia or autism (Hubbard et al., 2012; Straube et al., 2013a,b).

### Conflict of interest statement

The authors declare that the research was conducted in the absence of any commercial or financial relationships that could be construed as a potential conflict of interest.
